# Supplementary material for: Machine Learning Accelerates Crystallization for Structure Determination
Source: Angew Chem Int Ed Engl. 2026 May 4;65(25):e1218503. doi: 10.1002/anie.1218503 (PMC13266958; doi:10.1002/anie.1218503)

checkCIF/PLATON report

Structure factors have been supplied for datablock(s) lcz059

THIS REPORT IS FOR GUIDANCE ONLY. IF USED AS PART OF A REVIEW PROCEDURE FOR PUBLICATION, IT SHOULD NOT REPLACE THE EXPERTISE OF AN EXPERIENCED CRYSTALLOGRAPHIC REFEREE.

No syntax errors found. CIF dictionary Interpreting this report

Datablock: lcz059

Bond precision:	C-C = 0.0332 A		Wavelength=1.54184
Cell:	a=13.1618(1)	b=13.1618(1)	c=68.0701(13)
	alpha=90	beta=90	gamma=120
Temperature:	100 K		

	Calculated	Reported
Volume	10212.2(3)	10212.2(2)
Space group	P 65	P 65
Hall group	P 65	P 65
Moiety formula	C58 H22 Ag6 F36 N12 O4	2(C15 H3 Ag3 F18 N6), 2(C14 H8 O2)
Sum formula	C58 H22 Ag6 F36 N12 O4	C58 H22 Ag6 F36 N12 O4
Mr	2282.10	2282.09
Dx,g cm-3	2.227	2.226
Z	6	6
Mu (mm-1)	15.020	15.020
F000	6552.0	6552.0
F000’	6583.47	
h,k,lmax	16,16,86	16,16,84
Nref	14660[7393]	13110
Tmin,Tmax	0.124,0.223	0.361,1.000
Tmin’	0.020	

Correction method= # Reported T Limits: Tmin=0.361 Tmax=1.000
AbsCorr = MULTI-SCAN

Data completeness= 1.77/0.89 Theta(max)= 78.525

R(reflections)= 0.0802(12085) wR2(reflections)= 0.1600(13110)

S = 1.145 Npar= 1046

The following ALERTS were generated. Each ALERT has the format
test-name_ALERT_alert-type_alert-level.
Click on the hyperlinks for more details of the test.

Alert level B

PLAT342_ALERT_3_B Low Bond Precision on C-C Bonds .... 0.03321 Ang.

Author Response: Disordered structure.

PLAT971_ALERT_2_B Check Calcd Resid. Dens. 1.12Ang From Ag02 2.89 eA-3

Author Response: This Alert is due to presence of residual density in the presence of heavy metal atom (Ag).

PLAT971_ALERT_2_B Check Calcd Resid. Dens. 1.03Ang From Ag03 2.71 eA-3

Author Response: This Alert is due to presence of residual density in the presence of heavy metal atom (Ag).

PLAT971_ALERT_2_B Check Calcd Resid. Dens. 1.08Ang From Ag03 2.62 eA-3

Author Response: This Alert is due to presence of residual density in the presence of heavy metal atom (Ag).

PLAT971_ALERT_2_B Check Calcd Resid. Dens. 1.02Ang From Ag02 2.59 eA-3

Author Response: This Alert is due to presence of residual density in the presence of heavy metal atom (Ag).

PLAT971_ALERT_2_B Check Calcd Resid. Dens. 1.03Ang From Ag06 2.51 eA-3

Author Response: This Alert is due to presence of residual density in the presence of heavy metal atom (Ag).

Alert level C

PLAT042_ALERT_1_C Calc. and Reported MoietyFormula Strings Differ Please Check

```
              Calc: C58 H22 Ag6 F36 N12 O4
              Rep.: 2(C15 H3 Ag3 F18 N6), 2(C14 H8 O2)
PLAT090_ALERT_3_C Poor Data / Parameter Ratio (Zmax > 18) ........       6.96 Note
PLAT213_ALERT_2_C Atom F2               has ADP max/min Ratio .....        3.2 prolat
PLAT213_ALERT_2_C Atom F008             has ADP max/min Ratio .....        3.6 prolat
PLAT213_ALERT_2_C Atom F00C             has ADP max/min Ratio .....        3.2 prolat
PLAT213_ALERT_2_C Atom F00O             has ADP max/min Ratio .....        3.4 prolat
PLAT213_ALERT_2_C Atom F018             has ADP max/min Ratio .....        3.3 prolat
PLAT213_ALERT_2_C Atom F01B             has ADP max/min Ratio .....        3.7 prolat
PLAT213_ALERT_2_C Atom F01F             has ADP max/min Ratio .....        3.3 prolat
PLAT213_ALERT_2_C Atom F02G             has ADP max/min Ratio .....        3.4 prolat
PLAT213_ALERT_2_C Atom N01K             has ADP max/min Ratio .....        3.2 oblate
PLAT213_ALERT_2_C Atom C01H             has ADP max/min Ratio .....        3.3 oblate
PLAT213_ALERT_2_C Atom C026             has ADP max/min Ratio .....        3.1 oblate
PLAT220_ALERT_2_C NonSolvent   Resd 1  F   Ueq(max)/Ueq(min) Range        3.3 Ratio
PLAT250_ALERT_2_C Large U3/U1 Ratio for <U(i,j)> Tensor(Resd    1)        2.1 Note
PLAT906_ALERT_3_C Large K Value in the Analysis of Variance ......      7.142 Check
PLAT906_ALERT_3_C Large K Value in the Analysis of Variance ......      2.406 Check
PLAT971_ALERT_2_C Check Calcd Resid. Dens.  1.12Ang From Ag05          2.49 eA-3
```

Author Response: This Alert is due to presence of residual density in the presence of heavy metal atom (Ag).

```
PLAT971_ALERT_2_C Check Calcd Resid. Dens.  1.13Ang From Ag05          2.40 eA-3
```

Author Response: This Alert is due to presence of residual density in the presence of heavy metal atom (Ag).

```
PLAT971_ALERT_2_C Check Calcd Resid. Dens.  1.13Ang From Ag06          2.40 eA-3
```

Author Response: This Alert is due to presence of residual density in the presence of heavy metal atom (Ag).

```
PLAT971_ALERT_2_C Check Calcd Resid. Dens.  1.05Ang From Ag04          2.35 eA-3
```

Author Response: This Alert is due to presence of residual density in the presence of heavy metal atom (Ag).

```
PLAT971_ALERT_2_C Check Calcd Resid. Dens.  1.12Ang From Ag01          2.33 eA-3
```

Author Response: This Alert is due to presence of residual density in the presence of heavy metal atom (Ag).

```
PLAT971_ALERT_2_C Check Calcd Resid. Dens.  1.09Ang From Ag01          2.28 eA-3
```

Author Response: This Alert is due to presence of residual density in the presence of heavy metal atom (Ag).

```
PLAT971_ALERT_2_C Check Calcd Resid. Dens.  1.00Ang From Ag04          2.25 eA-3
```

Author Response: This Alert is due to presence of residual density in the presence of heavy metal atom (Ag).

```
PLAT977_ALERT_2_C Check Negative Difference Density on H01J      .      -0.53 eA-3
PLAT977_ALERT_2_C Check Negative Difference Density on H01M      .      -0.33 eA-3
PLAT977_ALERT_2_C Check Negative Difference Density on H02K      .      -0.40 eA-3
PLAT977_ALERT_2_C Check Negative Difference Density on H02P      .      -0.33 eA-3
```

Alert level G

```
PLAT002_ALERT_2_G Number of Distance or Angle Restraints on AtSite          6 Note
PLAT003_ALERT_2_G Number of Uiso or U(i,j)  Restrained non-H-Atoms         35 Report
PLAT083_ALERT_2_G SHELXL Second Parameter in WGHT  Unusually Large     166.91 Why ?
PLAT172_ALERT_4_G The CIF-Embedded .res File Contains DFIX Records          3 Report
PLAT178_ALERT_4_G The CIF-Embedded .res File Contains SIMU Records          2 Report
PLAT186_ALERT_4_G The CIF-Embedded .res File Contains ISOR Records          6 Report
PLAT188_ALERT_3_G A Non-default SIMU Restraint Value has been used     0.0100 Report
PLAT188_ALERT_3_G A Non-default SIMU Restraint Value has been used     0.0100 Report
PLAT242_ALERT_2_G Low    'MainMol' Ueq as Compared to Neighbors of       C020 Check
PLAT242_ALERT_2_G Low    'MainMol' Ueq as Compared to Neighbors of       C022 Check
PLAT242_ALERT_2_G Low    'MainMol' Ueq as Compared to Neighbors of       C025 Check
PLAT242_ALERT_2_G Low    'MainMol' Ueq as Compared to Neighbors of       C02B Check
PLAT242_ALERT_2_G Low    'MainMol' Ueq as Compared to Neighbors of       C02D Check
PLAT242_ALERT_2_G Low    'MainMol' Ueq as Compared to Neighbors of       C02J Check
PLAT242_ALERT_2_G Low    'MainMol' Ueq as Compared to Neighbors of       C02N Check
PLAT242_ALERT_2_G Low    'MainMol' Ueq as Compared to Neighbors of       C02O Check
PLAT242_ALERT_2_G Low    'MainMol' Ueq as Compared to Neighbors of       C02V Check
PLAT242_ALERT_2_G Low    'MainMol' Ueq as Compared to Neighbors of       C037 Check
PLAT242_ALERT_2_G Low    'MainMol' Ueq as Compared to Neighbors of       C038 Check
PLAT434_ALERT_2_G Short Inter HL..HL Contact F1       ..F00P     .       2.82 Ang.
                                              1+x,1+y,z  =      1_665 Check
PLAT720_ALERT_4_G Number of Unusual/Non-Standard Labels ..........        134 Note
              Ag01    Ag02    Ag03    Ag04    Ag05    Ag06    F007    F008
              F009    O00A    F00B    F00C    F00D    O00E    N00F    F00G
              F00H    F00I    O00J    N00K    N00N    F00O    F00P    C00R
              O00S    F00T    F00U    F00V    F00W    F00X    N00Y    F00Z
              N010    F011    F012    N013    C014    F016    F017    F018
              N019    F01A    F01B    N01C    F01D    F01E    F01F    C01G
              C01H    C01I    C01J    H01J    N01K    F01L    C01M    H01M
              F01N    N01O    F01P    C01Q    H01Q    C01R    F01S    C01T
              N01U    C01V    C01W    H01W    C01X    C01Y    C01Z    H01Z
              C020    C021    H021    C022    C023    C024    H024    C025
              C026    C027    C028    C029    H029    C02A    C02B    C02C
              C02D    C02E    H02E    C02F    H02F    F02G    C02H    C02I
              C02J    C02K    H02K    C02L    H02L    C02M    C02N    C02O
              C02P    H02P    C02Q    H02Q    C02R    H02R    C02S    F02T
              C02U    C02V    C02W    H02W    C02X    H02X    C02Y    C02Z
              C030    H030    C031    H031    C032    C033    C034    H034
              C035    H035    C036    H036    C037    C038
PLAT722_ALERT_1_G Angle   Calc      121.00, Rep      119.80 Dev...       1.20 Degree
              C034    -C02W    -H02W       1_555  1_555  1_555     #  210 Check
PLAT860_ALERT_3_G Number of Least-Squares Restraints ............        232 Note
PLAT883_ALERT_1_G Absent Datum for _atom_sites_solution_primary ..     Please Do !
```

```
PLAT912_ALERT_4_G Missing # of FCF Reflections Above STh/L=  0.600        111 Note
PLAT933_ALERT_2_G Number of HKL-OMIT Records in Embedded .res File          2 Note
               -5  8-75,  -5  8-74,
PLAT952_ALERT_5_G Calculated (ThMax) and CIF-Reported Lmax Differ.          2 Units
PLAT958_ALERT_1_G Calculated (ThMax) and Actual (FCF) Lmax Differ.          2 Units
PLAT969_ALERT_5_G The 'Henn et al.' R-Factor-gap value ..........      2.494 Note
              Predicted wR2: Based on SigI**2  6.42 or SHELX Weight 13.98
PLAT978_ALERT_2_G Number C-C Bonds with Positive Residual Density.          0 Info
```

```
   0 ALERT level A = Most likely a serious problem - resolve or explain
   6 ALERT level B = A potentially serious problem, consider carefully
  28 ALERT level C = Check. Ensure it is not caused by an omission or oversight
  30 ALERT level G = General information/check it is not something unexpected

   4 ALERT type 1 CIF construction/syntax error, inconsistent or missing data
  46 ALERT type 2 Indicator that the structure model may be wrong or deficient
   7 ALERT type 3 Indicator that the structure quality may be low
   5 ALERT type 4 Improvement, methodology, query or suggestion
   2 ALERT type 5 Informative message, check
```

Validation response form

Please find below a validation response form (VRF) that can be filled in and pasted into your CIF.

```
# start Validation Reply Form
_vrf_PLAT042_lcz059
;
PROBLEM: Calc. and Reported MoietyFormula Strings  Differ     Please Check
RESPONSE: ...
;
_vrf_PLAT090_lcz059
;
PROBLEM: Poor Data / Parameter Ratio (Zmax > 18) ........       6.96 Note
RESPONSE: ...
;
_vrf_PLAT213_lcz059
;
PROBLEM: Atom F2              has ADP max/min Ratio .....        3.2 prolat
RESPONSE: ...
;
_vrf_PLAT220_lcz059
;
PROBLEM: NonSolvent   Resd 1  F   Ueq(max)/Ueq(min) Range        3.3 Ratio
RESPONSE: ...
;
_vrf_PLAT250_lcz059
;
PROBLEM: Large U3/U1 Ratio for <U(i,j)> Tensor(Resd    1)        2.1 Note
RESPONSE: ...
;
_vrf_PLAT906_lcz059
;
PROBLEM: Large K Value in the Analysis of Variance ......      7.142 Check
RESPONSE: ...
;
```

```
_vrf_PLAT977_lcz059
;
PROBLEM: Check Negative Difference Density on H01J      .      -0.53 eA-3
RESPONSE: ...
;
# end Validation Reply Form
```

It is advisable to attempt to resolve as many as possible of the alerts in all categories. Often the minor alerts point to easily fixed oversights, errors and omissions in your CIF or refinement strategy, so attention to these fine details can be worthwhile. It is up to the individual to critically assess their own results and, if necessary, seek expert advice.

PLATON version of 17/09/2025; check.def file version of 17/09/2025

---

duplicate check

No duplication found

Datablock lcz059 - ellipsoid plot

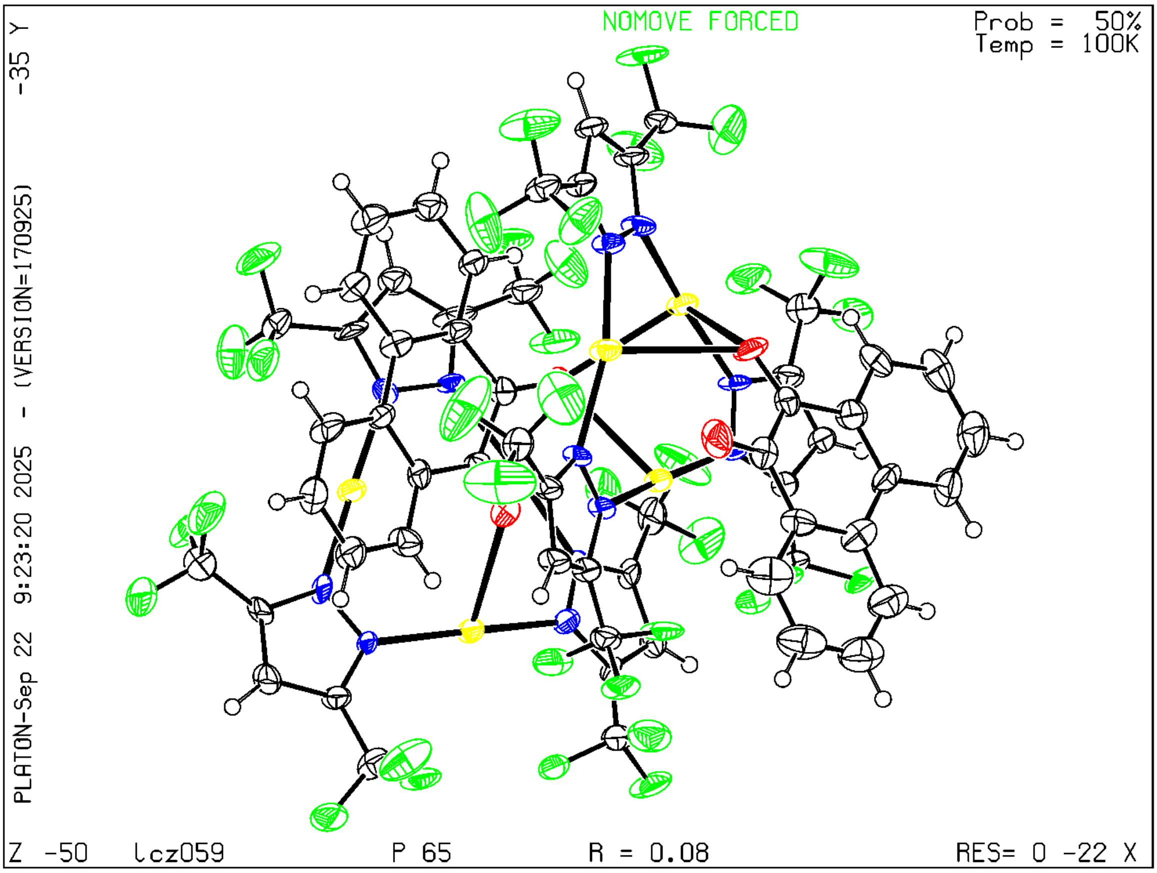

Supplement: Supplementary file 2 — Supporting File 2: anie72462‐sup‐0002‐DataFile.zip. [file ANIE-65-e1218503-s002.zip › supplementary_materials_crystallographic_data submit.zip!/cif_and_checkcif(1-114)/Ag3Pz3_59/Ag3Pz3_59_checkcif.pdf]
